# How digital design shapes political participation: A natural experiment with social information

**DOI:** 10.1371/journal.pone.0196068

**Published:** 2018-04-27

**Authors:** Scott A. Hale, Peter John, Helen Margetts, Taha Yasseri

**Affiliations:** 1 Oxford Internet Institute, University of Oxford, Oxford, United Kingdom; 2 Alan Turing Institute, London, United Kingdom; 3 Department of Political Economy, King’s College London, London, United Kingdom; Centre de physique theorique, FRANCE

## Abstract

Political behaviour increasingly takes place on digital platforms, where people are presented with a range of social information—real-time feedback about the behaviour of peers and reference groups—which can stimulate (or depress) participation. This social information is hypothesized to impact the distribution of political activity, stimulating participation in mobilizations that are increasing in popularity, and depressing participation in those that appear to be less popular, leading to a non-normal distribution. Changes to these platforms can generate natural experiments allowing for an estimate of the impact of different kinds of social information on participation. This paper tests the hypothesis that social information shapes the distribution of political mobilizations by examining the introduction of trending information to the homepage of the UK government petition platform. The introduction of the trending feature did not increase the overall number of signatures per day, but the distribution of signatures across petitions changed significantly—the most popular petitions gained more signatures at the expense of those with fewer signatories. We further find significant differences between petitions trending at different ranks on the homepage. This evidence suggests that the ubiquity of trending information on digital platforms is introducing instability into political markets, as has been shown for cultural markets. As well as highlighting the importance of digital design in shaping political behaviour, the findings suggest that a non-negligible group of individuals visit the homepage of the site looking for petitions to sign, without having decided the issues they wish to support in advance. These ‘aimless petitioners’ are particularly susceptible to changes in social information.

## Introduction

Online behaviour is characterised by non-normal distributions. From the number of hyperlinks pointing to a website [1], to the number of followers on Twitter [2] or the number of views of a video, many empirical distributions online appear to be heavy-tailed or exhibit a power law [3–6]. These distributions are not described well by their ‘typical’ or mean value as a large fraction of the points are very far from the mean. Thus, while the average candidate for parliament in the UK 2015 General Election had a mean 3,800 followers on Twitter, some candidates had none while others had over 100 thousand, with one having over one million followers [2]. This pattern does not resemble normal, bell-shaped distributions where extreme values are rare. The average American male is 180 centimetres, and while some males are taller or shorter no one is extremely far from this height [4]. While the power law is perhaps the most well-known heavy-tailed distribution, log-normal, exponential, and poisson distributions are also all heavy-tailed. The characterization of an empirical distribution as specifically following a power law is complicated (see [4] for a fuller discussion); so, we use the more general category of heavy-tailed in this article.

Heavy-tailed distributions are not exclusive to online behaviour—the distribution of phone calls received per person, the frequencies of words in novels, and the populations of cities, towns, and villages in various countries have all been characterised as heavy-tailed [4]. However, heavy-tailed distributions appear to be particularly common online [3–6]. Barabási et al. [7] suggest these distributions arise in online networks through the combination of two factors (1) continuous growth and (2) new entities attaching preferentially to existing entities that are already well connected (or popular). For example, creators of new webpages are likely to link to existing, popular webpages thus reinforcing the popularity of those webpages and leading to a distribution where a small number have many incoming links (are very popular) while most webpages have only a small number of incoming links. A tacit assumption of this model is that popularity is known or easily observed, which is much more likely to be true in a digital setting. Offline it may be difficult to observe what a large number of other people are doing. In contrast, many online platforms clearly indicate how many other people have already liked, followed, retweeted, signed, or otherwise undertaken various actions. This information on what others are doing or have done is known as social information and has a long history in social psychology [8, 9].

One reason then that we might expect heavy-tailed distributions to be particularly common online is the increasing ease with which social information is available. Applying the model of preferential attachment, a citizen looking to sign a petition would be most likely to sign an already popular petition, and someone looking to follow a politician (other than their representative) would be likely to choose one with many followers. Increasing the ease with which citizens can find the most popular petitions should lead to even more extreme values with a smaller number of petitions capturing an even larger proportion of signatures to all petitions. While the act of voting itself remains relatively free of social information as each citizen casts a ballot independently, political campaigning and mobilization—and indeed all activities that lead up to any political activity including voting—have undergone a dramatic increase in solicitation information [10]. The signing of petitions is an apt example: when petition signing was largely paper-based and distributed, it was difficult for someone considering signing a petition to know exactly how many other people had already signed it. Today, digital petition platforms prominently show the current number of signatories each petition has. Although we do not know the distribution of signatures across offline petitions (because in general, there is no record of the ones that failed), we do have substantial evidence from our earlier work to suggest that the distribution of signatures across petitions in the US and the UK is leptokurtic, rather than normal [10].

If social information is the key to the type of distributions that emerge online, then it will be key to understanding the nature of political mobilization, which increasingly takes place in digital settings (see [11]) and therefore under conditions of greater social information. In fact, there is evidence that distributions of political mobilizations are, like so many other digital activities, indeed heavy-tailed. So it could be that the influence of social information could ultimately help to explain the rapidity in the rise of new social movements and the fast-moving nature of the political and policy agendas in the twenty-first century [10]. Changes to the design of digital platforms provide a valuable opportunity to isolate and understand these social information effects on political behaviour, allowing us to test the hypothesis that social information drives non-normal distributions in political mobilization. Such changes create the conditions for a natural experiment: visitors to the site just before and after the change experience it as if it were random [12]. Under these conditions, changes in political behaviour before and after a re-design can be directly attributed to the re-design. This paper analyses the effect of one such change—the introduction of trending information on the homepage of the UK government petition platform—and its impact on petition signing. It uses data pertaining to Internet-based mobilizations around petitions, generated from the government petition platform in the UK, which was developed by the UK Cabinet Office for the incoming Coalition Government in 2010, launched in August 2011, and discontinued in 2015 ahead of the UK General Election that year (to be replaced by a new version hosted by Parliament). The data collected on the site since its launch provide digital trace data [13] on this popular form of political participation. Although the data is not mammoth in size, it does have many of the attributes of so-called ‘big data’: it is real-time, transactional, and represents an entire population rather than a sample.

In this article we first review previous research on social information and participation and relate it to online settings. Second, we provide some background on petitioning as a form of collective action and outline the distinctive features of the petition platform we examine here. Third, we present the petition data and analyse it with interrupted time series analysis and a regression discontinuity design. We discuss the key findings from the data, which suggest that users are influenced by the trending information. To conclude, we use site analytics data from the petition platform itself to explain the empirical observations and discuss the implications of our findings for research into political participation in digital settings (e.g., [14, 15]).

## Social information and digital media

The term social information originates from social psychology where social information processing refers to the study of the informational and social environments within which individual behaviour occurs and to which it adapts [8, 9]. Social information helps people decide what they are going to do with reference to a wider social group, which has the potential to activate people’s social norms. Potential participants construe this information as representing the behaviour of a ‘generalised other,’ or a social aggregate [16], and take it into account when they are deciding whether and how to participate. Social information has been shown to affect charitable giving and willingness to participate in public goods provision (see [17–22]). Economists have studied the effects of social information on people’s willingness to undertake pro-social behaviours, in particular in making charitable donations, where cooperation that is dependent on social information giving evidence of the contributions of others has been labelled conditional cooperation. Social information may crowd out contributions or crowd them in, with evidence pointing to the latter because of conformity, social norms, and reciprocity (see [21]). People are more likely to contribute to a campaign if they are provided with information that other people are also doing the same, and this effect increases with larger numbers of additional participants [21, 23]. Researchers have also shown that people are likely to increase their contributions (by donating more money, for example) if they know that other people are increasing the size of their commitments [22, 24]. In sociology and political science, experimental studies have uncovered the importance of social information on people’s willingness to contribute to public goods by undertaking activities such as recycling [20, 25, 26] and voting [27, 28]. Social information provides a powerful signal of viability for a mobilization, evidence of whether or not it has reached or will reach critical mass [29] and hence the potential benefits of joining, thereby altering the incentives of individuals to participate.

When social information is provided precisely and in real-time, as facilitated by social media, we expect that these effects on mobilization will be all the more profound and intractable. For example, we might expect to see in a political context the same kind of effects observed in cultural markets, where Duncan Watts and colleagues [30, 31] carried out a series of experiments to show how changes to real-time information feedback about cultural artefacts (songs) changed the way that people viewed their qualities, leading popular songs to become more popular and unpopular ones less so. In the context of online mobilization, social information if presented as a large unit, such as a million signatures, can act like a nudge [32], which can encourage participation.

Furthermore, social media and mobile technologies reduce the transaction costs of political participation: they facilitate ‘micro-donations’ of time, effort and money towards political causes, ease the making of small monetary donations to political causes (texting a keyword to donate £3 to a disaster relief effort, for example), and extend the lower end of the ‘ladder’ of participation. As the costs of participation fall, the cost-benefit analysis facing someone deciding whether to participate changes [11], and social information is likely to be proportionately more important an influence when deciding whether to make such a micro-donation, as other influences diminish. Indeed, recent research investigating the relationship between Internet use and participation has shown that where costs of participation are very low, interest in politics reduces in importance as a causal factor of participation and that skilled Internet users do not need to be motivated or interested in politics in order to participate online [14].

In the next section, we outline the changing form of political participation in online settings, in particular the rise of petitioning, which forms the context of our study.

## Petitions as political mobilizations

Signing petitions has long been one of the more popular political activities, leading the field for participatory acts outside voting, and with other social benefits such as civic mindedness [33] ascribed to it in addition to its potential to bring about policy change. In the UK, the right to petition the King goes back to medieval times. Petitions were widely used by the 18th century and were a key mechanism in the campaign for parliamentary reform in the early 19th century [34], when petitioning was a popular activity in the US [35, 36]. After a period of decline through the 20th century, petitioning received renewed interest in the 21st, with the availability of electronic petitions that could be created, signed, and disseminated on the Internet and, more recently, social media. Although there are start-up costs in getting to know the platform for people who initiate petitions, they can find supporters easily, rather than having to canvass them door-to-door or approach people in the street. For those wishing to sign petitions, the search costs are far lower: they may sign a petition instantly on receipt of an email or post on a social networking site, or go to one of the large number of petition platforms and look for a petition to sign, rather than having to wait until they encounter a petition in the course of other activities. Furthermore, every petition signer is now potentially also a petition organizer given the ease with which petitions can be disseminated to one’s contacts via social media. With these reduced transactional costs and the ease of coordination, petitions have been very popular in the age of social media, one of a growing portfolio of Internet-based democratic innovations [37]. Both governments and NGOs, such as Avaaz [38] and 38 Degrees, have made widespread use of petitions and accordingly have received accolades for their democratic contribution by academic commentators [39, 40]. A growing number of governments have implemented petition platforms, notably the UK, the US and Germany. While petitions create no binding obligations for governments, several petitions have had large policy impact. One notable petition on road pricing in the UK received 1.8 million signatures in 2007 and is widely regarded as a key reason for the government’s policy reversal. A petition in the United States on cell/mobile phone unlocking lead to the passing of new legislation ensuring consumers on all networks can unlock their phones [41]. Political scientists have looked at the German petition platforms [42, 43] and UK petition platforms [34, 44, 45], and others have examined petitioning behaviour and activism, in particular exploring whether a small number of highly active people are driving growth in signatures or whether activity is more distributed across society at large [46–48].

The version of the petition platform that we investigate here was set up by the UK Cabinet Office and the Government Digital Service in 2011, when it replaced an earlier site operated by No. 10 Downing Street website, which received more than 8 million signatures from over 5 million unique email addresses over the course of its lifetime from November 2006 until 4 April 2011 [49]. The Cabinet Office launched the new petition site in August that year, initially on the direct.gov portal (which eventually became the gov.uk portal in the autumn). Each petition created on the site represents a focal point for a possible mobilization of potential signatories, which varied from less than a handful to hundreds of thousands.

Our previous work examined large-scale data generated from this UK platform to understand the ecology of these petition-mobilizations, and analysed the growth curves of successful petitions, identifying early, rapid growth as being critical for any kind of success [10, 50] and highlighting the heavy-tailed or leptokurtic distributions we discussed in the introduction. Related work has also analysed the distribution of time between signatures to petitions [51]. This study is the first to take an experimental approach to test the effect of alternative platform designs on petitioning behaviour in the wild, using large-scale data generated from the platform itself. By testing the effect of a design change that introduced trending information, we contribute to understanding of how different presentations of social information feed into the distribution of political mobilization in digital settings.

## Social information and popular petitions

**Fig 1:**
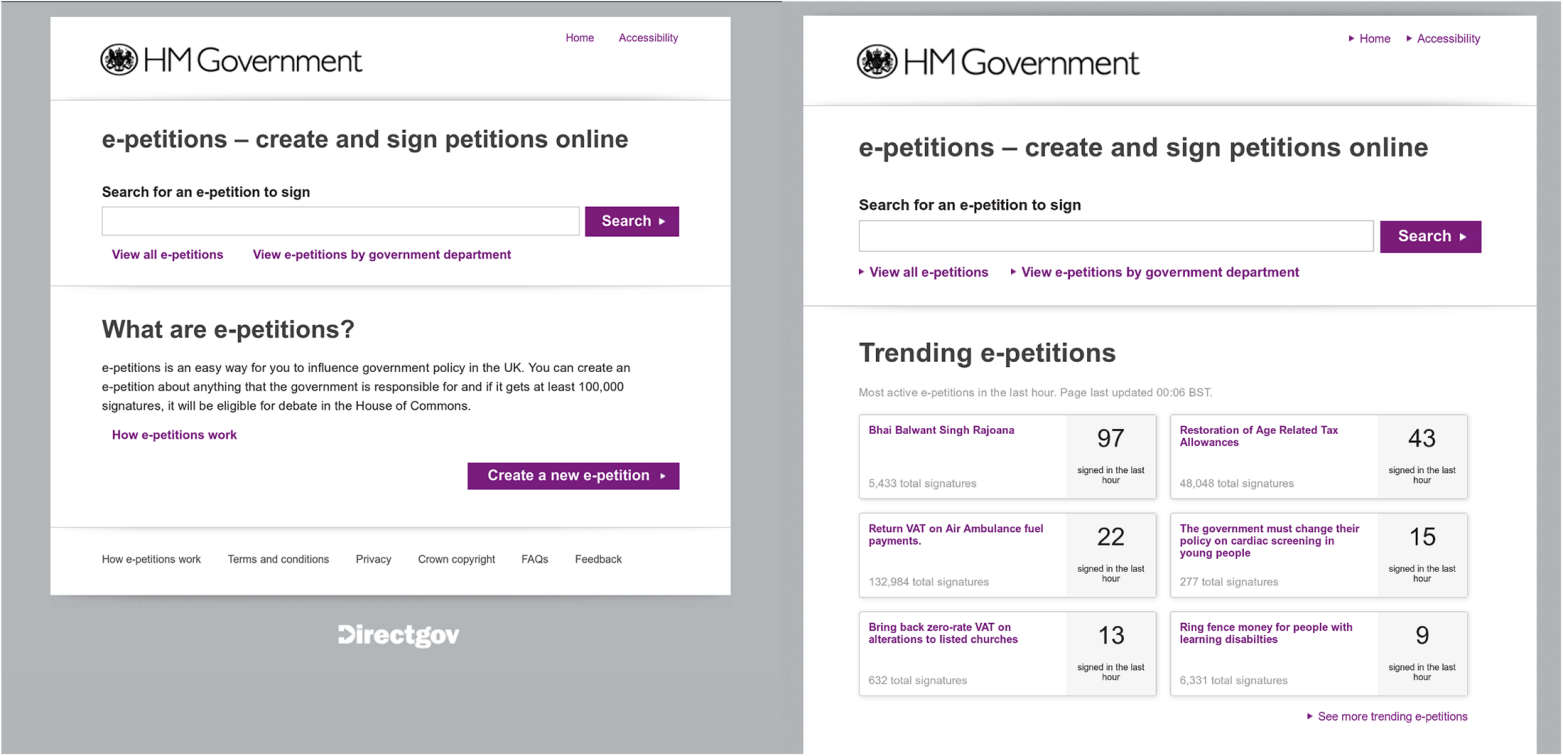
Left: A screenshot of the homepage on 27 March 2012 (no trending information). Right: A screenshot of the homepage on 30 March 2012. A list of trending petitions has been added. Both screenshots are from the Internet Archive.

All versions of the UK government petition platform have displayed social information, in terms of the number of other people who had signed any given petition. It has always been possible to sort the list of petitions by popularity that is, by the number of signatures, and indeed popularity has been the default sorting order since the Cabinet Office version of the site launched. In March 2012, social information was emphasised when a change was made to the site to show trending petitions on the homepage: a list of the six most popular petitions in terms of total number of signatures within the last hour (Fig 1). This change meant that these petitions were flagged for attention to any visitor to the homepage, with social information automatically provided—regardless of whether the visitor sought it out or not—and acting as a potential driver for signature growth. Therefore, this change to the platform provided the opportunity to test the hypothesis that social information of this kind (that is, an indicator of popularity and rate of change, given that the number of signatures in an hour indicates movement on a petition) acts to encourage participation for those petitions for which it is provided. We would expect that those petitions shown on the homepage as trending to have received disproportionately more signatures compared to those not shown.

Such expectations about feedback depend on the shape of social responses in collective action on the Internet. From the theoretical discussion reviewed earlier, we consider two alternative hypotheses:

H1 Trending information will lead to more overall signatures on the petition platform.
H2 Overall signatures on the petition platform will remain constant, but trending petitions will receive more signatures at the expense of non-trending petitions.

We can test these hypotheses because, as indicated above, we have collected all the signatory data for the platform before and after the change. This date-range creates a natural experiment on the platform, allowing us to examine behaviour before and after the change in a regression discontinuity design.

## Data and methods

Online petitions are created, disseminated, circulated, and presented online. They are regularly disseminated online via social media platforms, particularly Facebook and Twitter. Although policy-makers may discuss responses in offline contexts, such responses are disseminated online. So both successful and unsuccessful mobilizations that form around petitions leave a complete digital audit trail of the real (rather than reported) actions of the entire population of signers of each petition, however large or small. In contrast, paper-based petitions left a rather incomplete record given that unsuccessful petitions not presented to the government or legislature were unlikely to be recorded, although successful ones can now be transcribed to digital form and analysed (see [36] for such an analysis of anti-slavery petitions in the US).

When the Cabinet Office petition site was launched in August 2011, we programmed an automatic script to scrape it every hour, recording the number of overall signatures to date on each active petition. In addition, we collected the title, text, launch date, and other attributes of each petition. Our dataset contains hourly data points for all the petitions (19,789) submitted to the site between 5 August 2011 and 22 February 2013. We also have anonymous website analytics data from December 2012 to April 2014, which is after the site redesign in March 2012. These data made it possible to examine the different patterns of growth in the 20,000 mobilization curves that we have data for and identify the distinctive characteristic of those mobilizations that succeed and those that fail. Such an analysis can tell us a great deal about petitioning and indeed, about the nature of collective action itself in a digital world.

Our previous work has analysed data from multiple UK government petition platforms to show that most petitions fail [50, 52]. Consistently across the datasets, only 5 per cent of petitions obtained 500 signatures (sufficient to gain an official response on the first platform). On the second platform, which we study here, 4 per cent received 1,000 signatures or more, 0.7 per cent attained the 10,000 signatures required to receive an official response, and 0.1 per cent attained the 100,000 signatures required for a parliamentary debate. We also found that the first day was crucial in achieving any kind of success on both platforms [52]. Any petition receiving 100,000 signatures after three months needed to have obtained 3,000 within the first 10 hours on average. To quantify further the fast decay in the popularity of the petitions, we modelled the number of signatures over time using a modulated multiplicative process and observed that the modulating parameter, which we labelled the ‘outreach factor,’ decayed very rapidly, reducing to 0.1% within 10 hours of the launch of a petition [52].

As noted above, in March 2012, the UK Cabinet Office introduced a change to the petition site that altered the information environment of prospective petitioners, by introducing a ‘trending petitions’ facility on the homepage, providing potential signatories with a new kind of social information about which petitions were currently popular and how many other people had signed each of these petition. We assume that such a change is exogenous to political participation itself; so, the time directly before and after the change is as if random. The fact that we captured data from this site both before and after this change provides us with a natural experiment (avoiding ‘bundling’ problems [12]) whereby we can test the effect of this change using a variety of methods, including interrupted series and regression discontinuity design.

## Results

**Fig 2:**
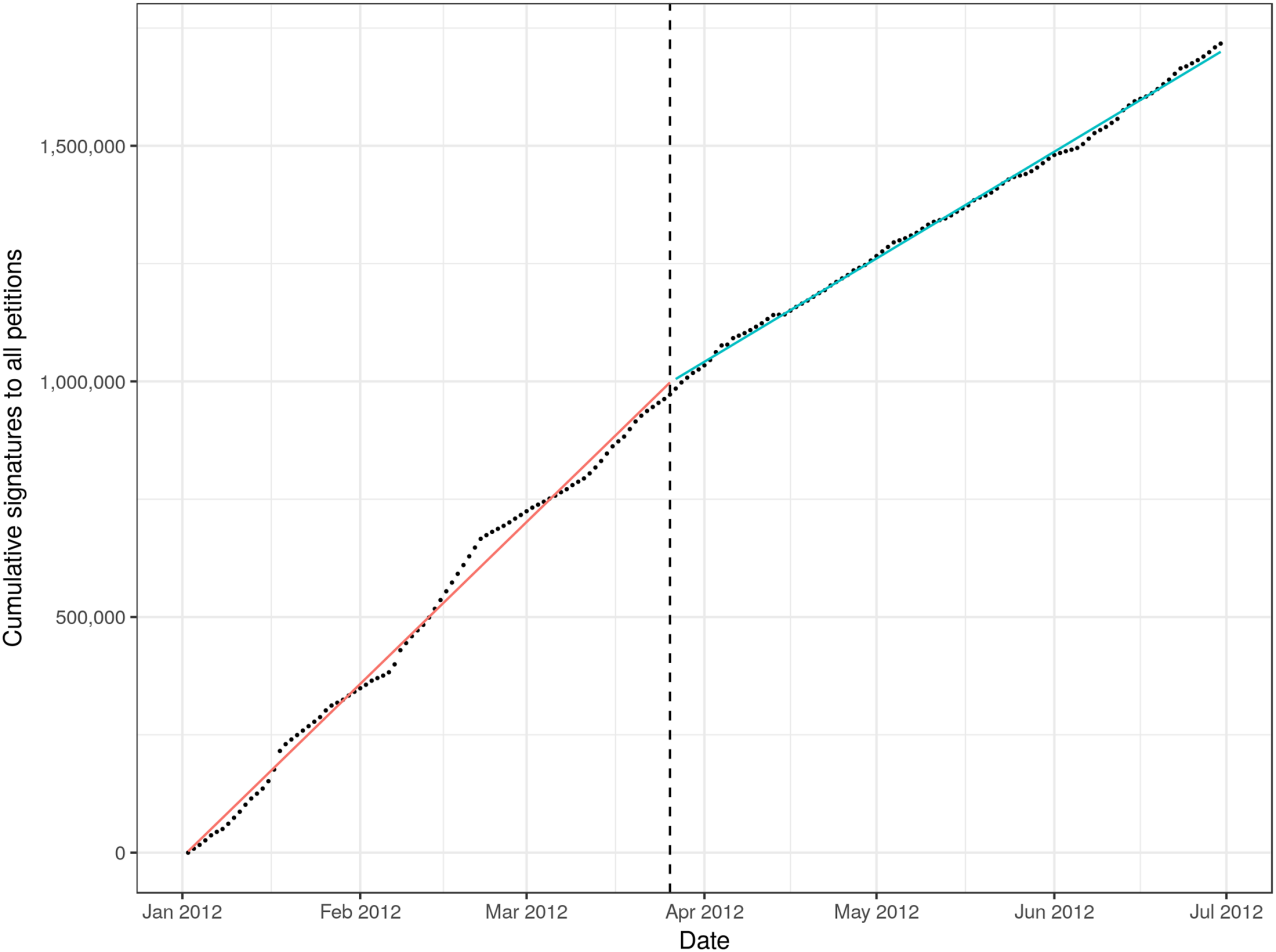
The cumulative number of signatures to all petitions on the platform. A piecewise linear function achieves the best fit with a break point at 26 March 2012 (vertical dashed line), which aligns approximately with the introduction of the trending information on the homepage. Contrary to H1, the number of signatures per day did not increase after the introduction of the trending information.

First, we tested the hypothesis (H1) that the trending facility would increase the overall number of signatures on the site, by attracting more visitors to the most popular petitions. In Fig 2, we plot the cumulative number of signatures to all petitions on the site over time from 1 January 2012 to 1 July 2012. We fit a piecewise linear regression model with one unknown break point following [53, 54] and find the best fit is achieved with a break point on 26 March 2012, which approximately aligns with the introduction of the trending petition feature on the site. The estimated slope for the model before the change is 11,846 signatures per day (standard error 77), while after the change the slope is estimated at 7,308 signatures per day (standard error 64). Contrary to our first hypothesis (H1), the trending information did not lead to more overall signatures on the platform. We found the results were sensitive to the exact start and end dates within which we examine the data. Looking at larger windows starting before November 2011 or ending after July 2012 showed the best location for the breakpoint at earlier/later dates. Regardless, the results never indicated an increase in the number of daily signatures as a result of the trending information.

**Fig 3:**
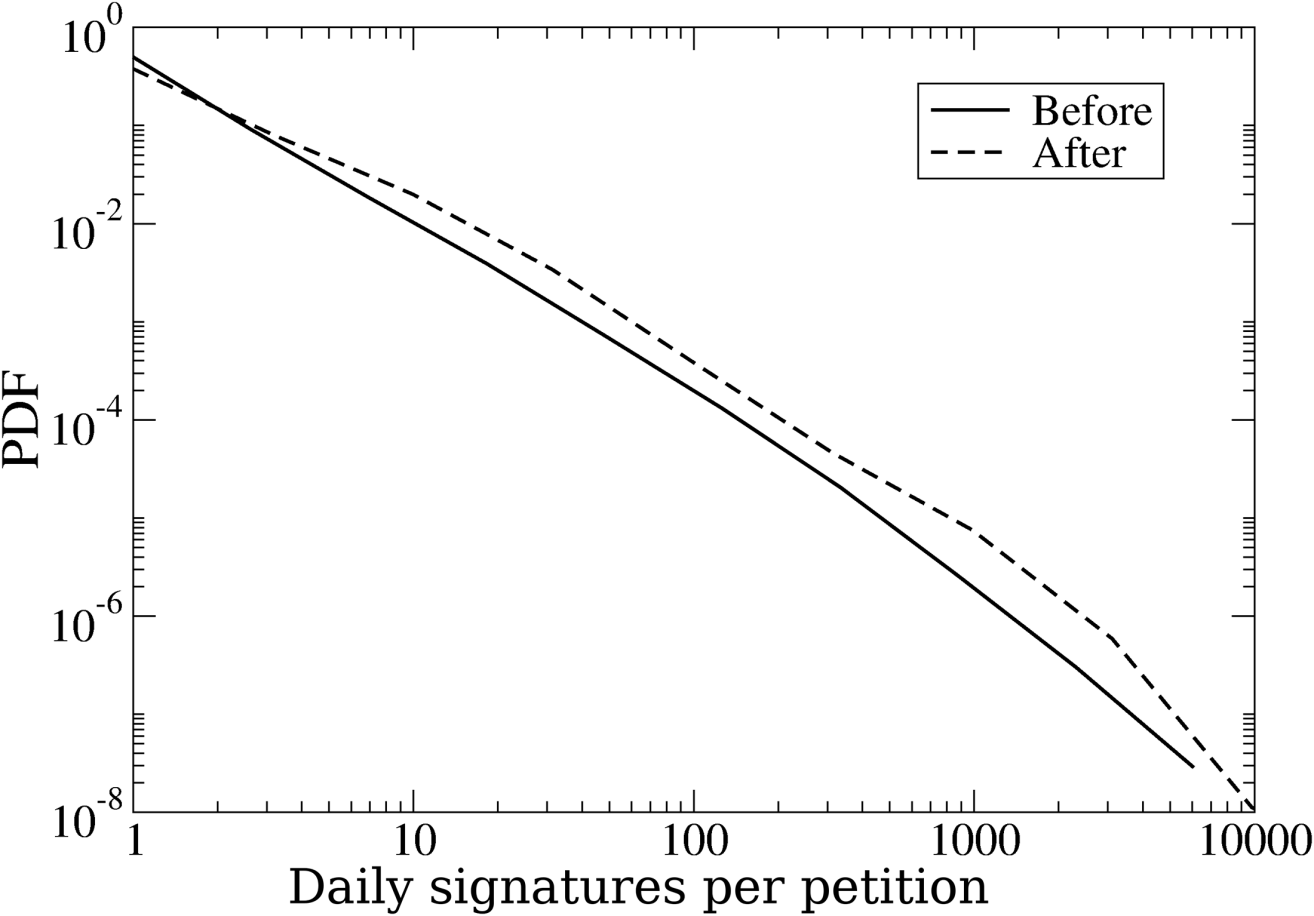
The daily distribution of signatures to petitions before and after the introduction of the trending facility.

If the introduction of the trending petitions facility did not increase the overall number of signatures on the site, did it change the distribution of signatures across petitions as predicted by hypothesis H2? Do the most popular petitions that appear in the trending box become more popular than they would have been had the facility not been introduced? We attempted to answer these questions by quantifying the distribution of the daily signatures to petitions (Fig 3). It is already evident from Fig 3 that the distribution becomes more skewed with more extreme values at the right end.

**Fig 4:**
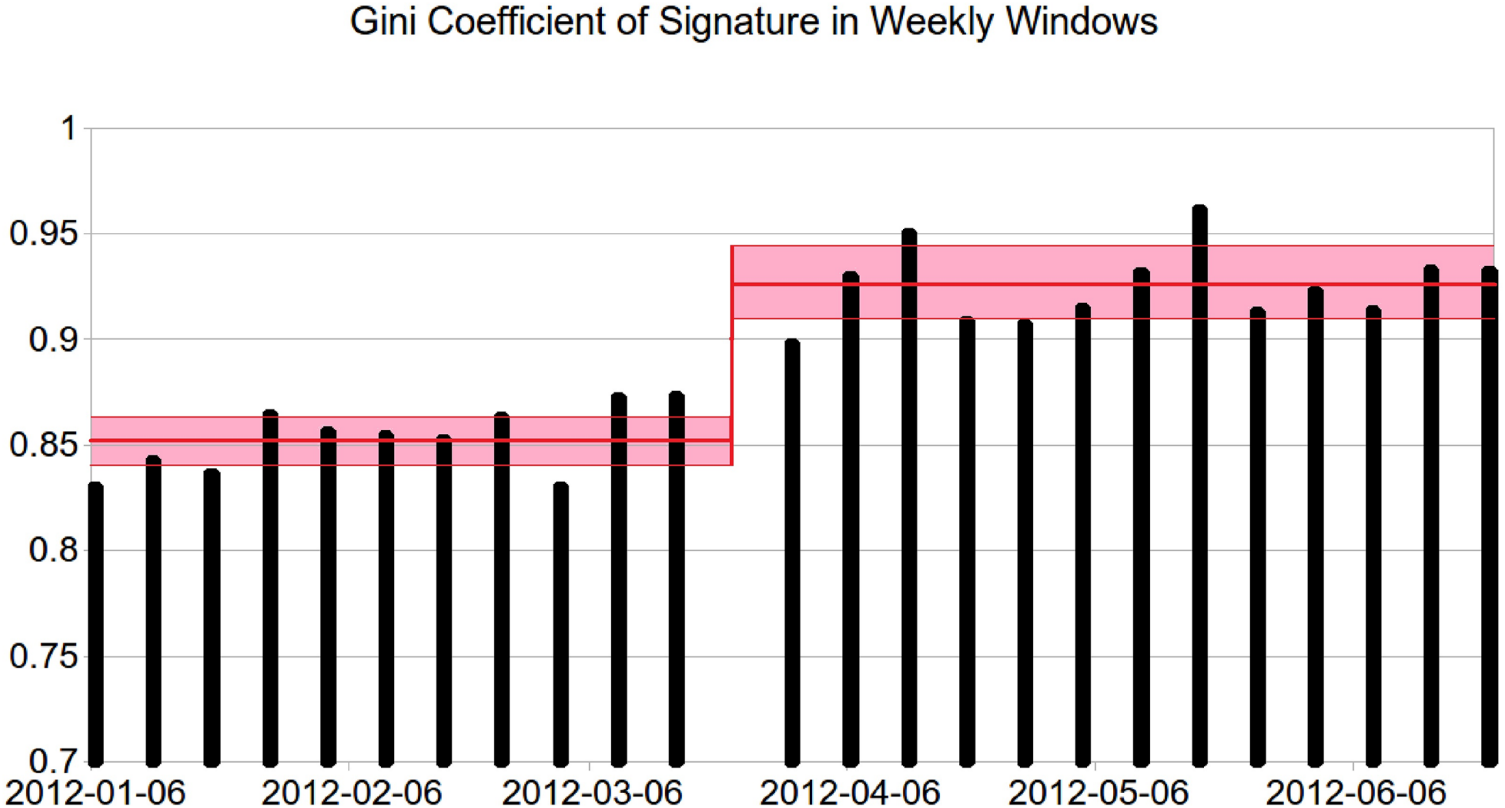
The effect of a change in design on the Gini coefficient.

To investigate the difference between the distributions of signatures further, we calculated the Gini coefficients for the distribution of signatures over petitions for one-week time windows. The weekly coefficients are shown in Fig 4. The thick red line shows the average over periods of three months before and after the change and the width of the pink area corresponds to the standard deviation between the weekly coefficients in each period. After the trending petitions facility was introduced, the Gini coefficient increased significantly from 0.85 to 0.92 (*p* < 0.0001 with Student’s t-test).

We can conclude then that the introduction of trending petitions on the homepage changed the distribution of signatures across petitions. The increase in the Gini coefficient indicates that signatures were more concentrated on a small number of petitions after the design change than they were before. The overall number of open petitions was always increasing (as petitions are open one year and at the time of the change the site was less than one year old). That said, we know that attention is most focused on new petitions, and the number of new petitions being created was decreasing during this time. This is probably the decay in the novelty of the website. There was a huge spike in activity right as the site launches (autumn 2011) and then a gradual decay in petition creation through the time period we study (spring 2012). We are interested in the effect of the interruption to these long-run trends.

The change in the Gini coefficient indicates that the distribution of signatures across petitions changed. Presumably the trending petitions captured more signatures at the expense of non-trending petitions, but the Gini coefficient cannot measure this directly. We now test this hypothesis directly with a regression discontinuity (RD) design, which is where a regression on the outcome using observations around the cut point can estimate the impact of the variable of interest [12]. The top trending petitions were shown in a 2-column, 3-row grid with the option to click a link to expose petitions in positions 7–12 (Fig 1). In order for the RD design to be appropriate, it must not be possible for any person to control which petitions trend or the positions at which they trend. Our data satisfies this condition as each user could only sign a petition once, and users did not know how many others would sign the same petition or another petition. We compare the signatures petitions in adjacent positions receive while trending on the homepage, and the important difference in outcomes is between petitions being placed in different positions (something any individual person would have little control over).

Our analysis here differs from a classic RD design in three ways. First, the number of signatures a petition receives is a discrete variable; so, we use the generalization of the RD design for discrete values used in Narayanan et al. [55]. Second, the position of a petition is determined by the number of signatures in the hour prior to trending while we measure the number of signatures in the hour while trending, and we cannot assume independence between these values and thus seek to compare the change in growth for each petition above its mean. Finally, we only observe trending petitions once an hour while the list is updated continuously in real time.

**Fig 5:**
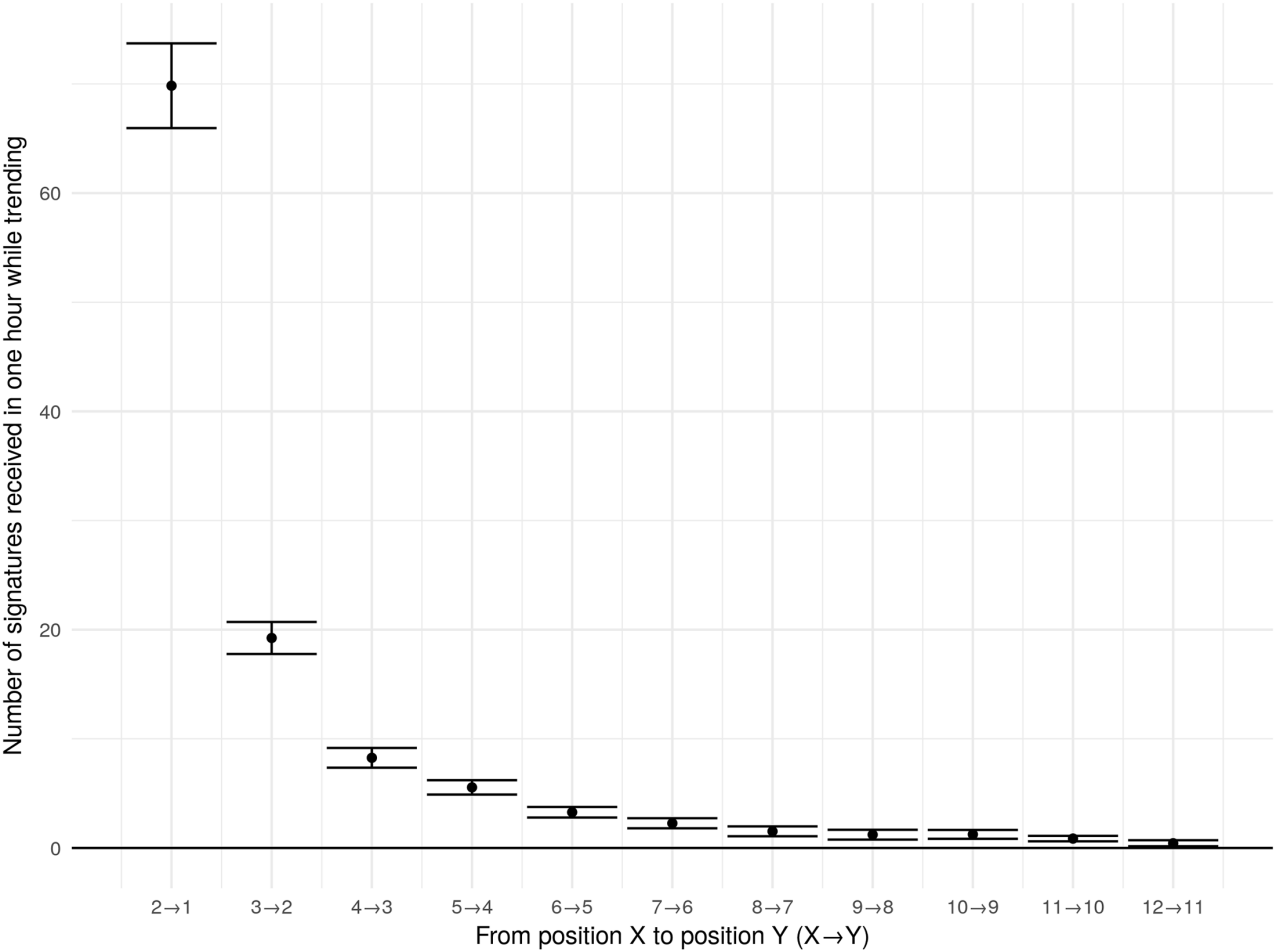
Comparisons of raw signatures in adjacent positions.

Fig 5 shows the difference in the raw number of signatures petitions in adjacent positions receive within one hour. The graph shows 95% confidence intervals in the difference in the raw number of signatures petitions in adjacent positions receive while trending. Petitions in position one, for instance, receive 66–74 more signatures in the hour while trending than petitions trending in position two. This effect diminishes rapidly for lower positions.

**Fig 6:**
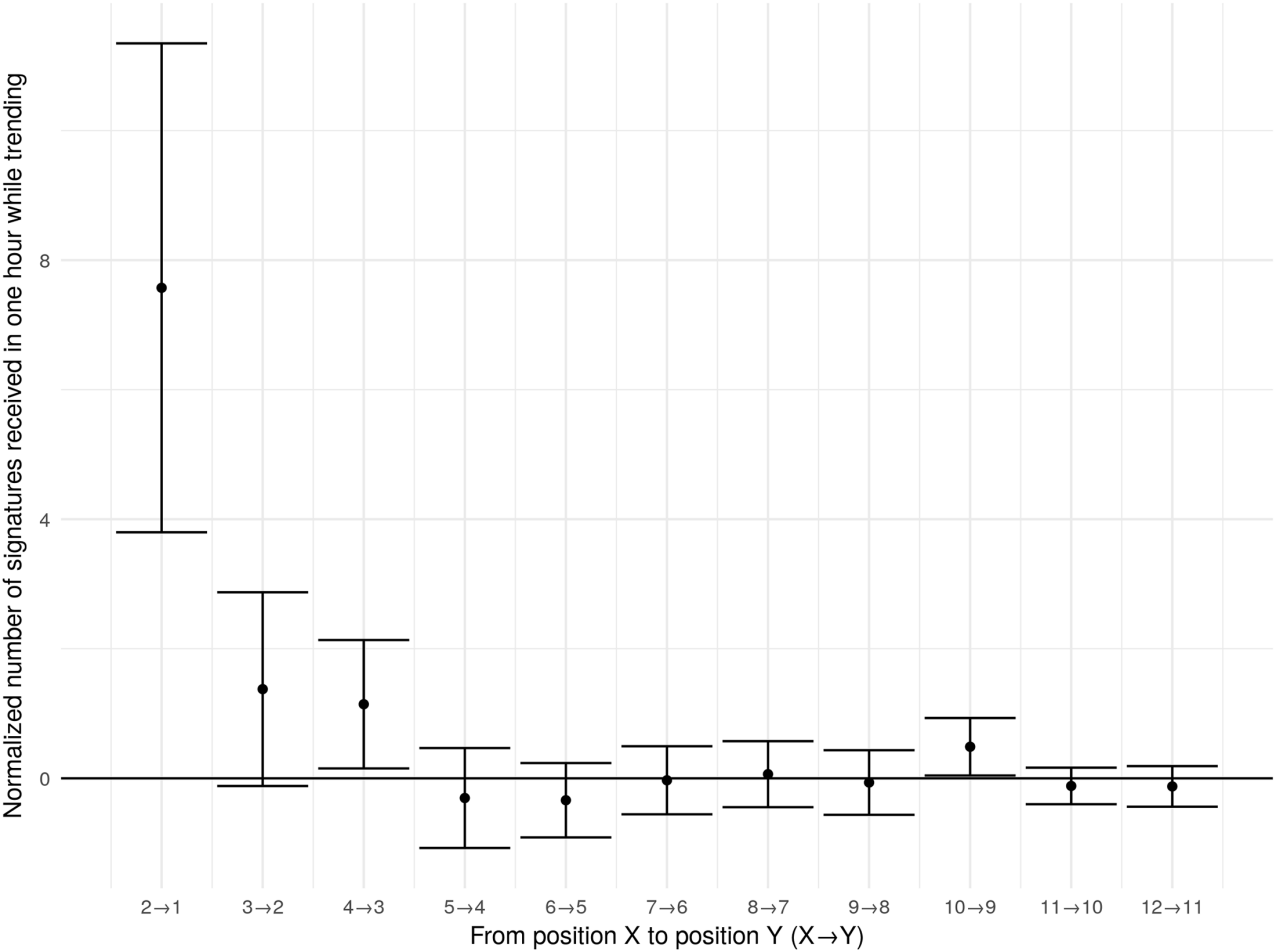
Comparisons of difference from 18-hour means in adjacent positions.

This analysis, however, does not take into account the differences among individual petitions. The petition in position one is in that position precisely because it is receiving more signatures than the petition in position 2. To control for these differences, we calculate the mean number of signatures for each petition over a number of hours before it trends. Fig 6 compares the number of signatures each petition receives above its 18-hour mean for adjacent positions. The graph shows 95% confidence intervals in the difference between petitions trending in adjacent positions simultaneously, but first subtracts from each petition its mean number of signatures for the 18 hours prior to trending in order to control for the individual variation of petitions. The results are stable for windows of other sizes larger than 18 hours.

Within the limitations detailed above, our analysis shows that the trending petitions facility did concentrate attention and signatures on trending petitions, but also that it did so differently depending on the positions of the petitions. That is, we find that the petition trending in the first position received 3.8 to 11.3 more signatures above its per hour mean than the petition trending in the second position. Similar, a move from position 4 to position 3 was associated with 0.2 to 2.1 more signatures above the petition’s per hour mean. The differences between other adjacent positions are not statistically significant.

While the trending facility did concentrate signatures, it did so only for petitions in the first few positions. It may seem strange that there are significant effects for moving from position 2 to position 1 and from position 4 to position 3, but not for moving from position 3 to position 2. We suspect the two-column layout is responsible for this oddity. Human-Computer Interaction studies using eye-tracking suggest users of search engines, for example, fixate on the results in the top position and then skim down the left side of the page (at least for users with left-to-right languages) [56]). This tendency to skim down the left side of the page means petitions in the left column (positions 1, 3, and 5) could have stood out more than petitions in the right column (positions 2, 4, and 6) and likely explains why a move from position 3 (on the left) to position 2 (on the right) had no significant effect.

Overall, our results provide a field test for the provision of social information and find that the trending petition information concentrated attention to the top few ranked petitions. We expected to find a significant difference between petitions in positions 6 and 7, because position 6 appeared in the bottom right of the trending petition information while viewing position 7 required a further click to ‘see additional trending petitions.’ However, attention is so concentrated on the first few positions that we find no significant difference between petitions in positions 6 and 7.

The analysis of positions in combination with that of overall signature growth and the Gini coefficient show that the addition of the trending petitions facility resulted in trending petitions receiving more signatures and that these signatures came at the expense of signatures to other petitions on the site (thus the change in the Gini coefficient while the overall number of signatures per day did not increase), validating H2. Simply put, the rich get richer and the poor get poorer.

**Fig 7:**
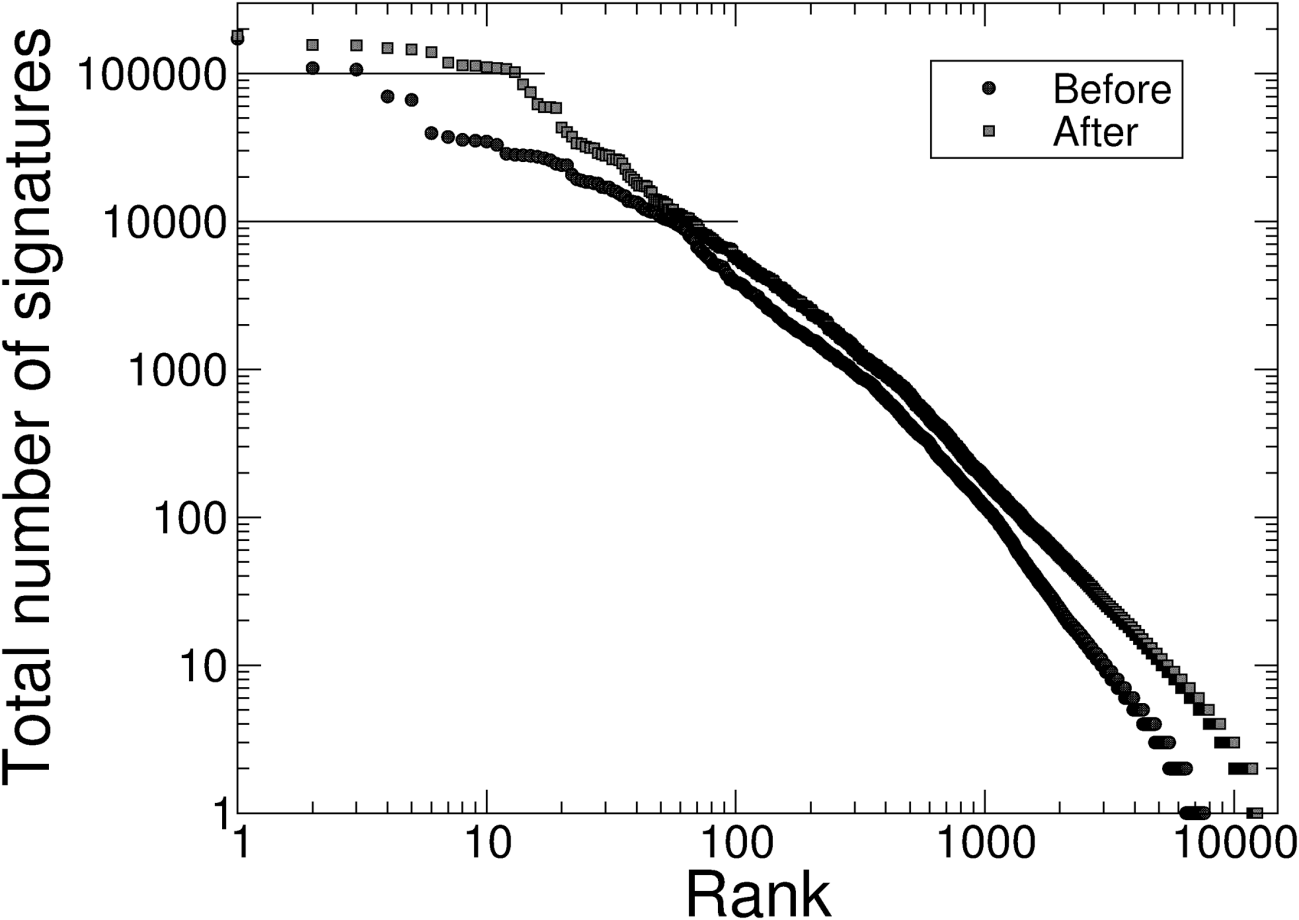
Comparisons of the total number of signatures that petitions received before and after the introduction of the trending petitions facility.

Such a result shows evidence of the heavy-tailed distributions that crop up so often in Internet-based distributions, where social information is available about which online initiatives are popular. In this case, however, the implications of this result are somewhat surprising. It suggests that some people come to the homepage of the petition platform looking for petitions to sign, or that they come to a specific petition on the site and then move on to the homepage looking for other petitions that interest them. This corresponds to Wright’s finding of ‘super-participants’ [45] and Huang et al.’s findings with ‘power users’ [47] who sign many petitions. These people have a zero-sum attention capacity: they will sign a certain number of petitions, but this number does not appear to have increased after the trending facility was introduced; so, if a particular petition attracts their attention, they will sign that one at the exclusion of another that they otherwise might have signed. As a result of the concentration of signatures, the number of the most successful petitions (petitions above the 100,000 signatures threshold) increased from 3 to 14 after the introduction of the trending information as shown in Fig 7.

## Understanding the impact of social information

This evidence that people are coming to petition sites just to ‘find something to sign’ (rather than coming to sign a petition on a specific issue) suggests a general desire for political engagement—the ‘aimless surfing’ identified by Borge and Cardenal [14]—without a firm view as to what the engagement should be about. These individuals are not completely aimless surfers, however, because they know what they want to do, that is, to sign a petition. To understand this observation further, we use anonymous analytics data for December 2012 to April 2014 provided by the UK Government Digital Service. However, we cannot match the analytics data (which relates to user visits to the site) with the petition data (which relates to petition signatories), and in any case the analytics data does not include the time that the platform was changed.

First, we look at the overall traffic sources to the website. Close to 40 per cent is directed from Facebook (two thirds of which is from the mobile version), 10 per cent from Twitter, and 10 per cent from Google organic search results. We analysed the flow of the users in order to investigate how many users saw the trending petitions information on the homepage and from where those users came. To check the stability of the forthcoming measurements, we repeated them for a shorter period of 6 months starting from December 2012. The results are all broadly similar with differences only in the decimal digits, and we therefore conclude that the results are stable at the reported precision.

Overall, about 3.5 per cent of all the visits to the website start at the homepage displaying the trending petitions information. If we look at the traffic sources of this 3.5 per cent, we observe a very different pattern compared to the overall visits: only 6 per cent of this 3.5 per cent is sourced from Facebook (equally from mobile and desktop) while Twitter is only responsible for 2 per cent of this direct traffic to the front page. About 44 per cent of the visits starting at the homepage originated from Google and 30 per cent of them were direct visits (by users who had typed the web address of the petition site directly into their browsers, bookmarked the page, or clicked a link in an email). That many users starting at the homepage came from Google is not surprising, considering that the first search result in Google for the keyword ‘petition’ directed to this page for users in the UK.

These observations explain the previous findings on the effect of the design change: a considerable number of users just go to the website directly or search for the petition website on Google, without aiming at a certain petition. We may think of these users as aimless petitioners. Apart from this group who visit the homepage and the trending petition information directly, almost 10 per cent of all the first clicks within the website (that is, by users who arrived at a page other than the homepage on the site) lead users to the homepage (with the trending petition information). Eventually, a consistent number of site visits include viewing the trending petition information on the homepage, including some users who sign a petition and then visit the homepage. Overall, this led to 2.35 million visits to the homepage (1.40 million unique visitors) out of the 63.6 million page views on the site overall (47.1 million unique page visits) during the period of traffic analysis. There were about 5.9 million visit sessions with at least one interaction with the site content. We estimate that about 40 per cent of all these sessions passed through the homepage at some point.

Using these numbers it is possible to estimate the theoretical maximum amount of change in the Gini coefficient. The first order approximation of the amount of change in the Gini coefficient Δ*g*, is equal to (1 − *g*_0_)(Δ*N*/*N*) where *g*_0_ is the Gini coefficient without social information and Δ*N*/*N* is the fraction of influenced visitors. By plugging in the values of *g*_0_ = 0.85 and Δ*N*/*N* = 0.40 (we assume all users to the homepage are influenced by the trending information and sign one of the top few petitions), we find that the maximum theoretical value for Δ*g* is 0.06.

Thus, the theoretical maximum amount of change in the Gini coefficient of the signature distribution that we would expect to see due to the social influence is about 6 per cent, which is in agreement with the amount of change in the Gini coefficient that we observed in the signature data (0.07), indicating that the effects of the local treatment is very strong and almost every user receiving the social information is influenced. (We remind the reader that the traffic data and the petition signature data come from different time periods and the theoretical value is only an approximation).

The results clearly show that an increase in the salience of social information increases the skew of the distribution. Thus part of the pervasiveness of heavy-tailed distributions on Internet-based platforms may be due to the prevalence of social information on such platforms. As discussed further in the next section, the Gini coefficient on the petition platform was high to start likely due to social information elsewhere on the petition platform as well as on the many social media platforms through which petitions are disseminated.

## Conclusion

In conclusion, we have analysed new data on political behaviour to investigate social information effects in a digital environment, suggesting that some citizens are using the Internet for generalized (rather than issue-based) civic engagement and showing how the design of Internet-based participation platforms can have significant effects on individuals’ political behaviours. We have examined the results from a natural experiment, where the effect of platform design changes can be observed and explained. We have verified the importance of social information by showing that information about the participation of others—trending information—can have an important effect on those who are exposed to it. Now that so much of political participation takes place in digital contexts where people are invited to make low-cost ‘micro-donations’ of time and effort to political causes as they go about their daily lives on social media [10], social information is more abundant, and we can expect that social information effects of this kind will be more prevalent than in offline contexts where decisions to participate tend to be larger, more ‘lumpy,’ and harder to influence (and therefore less likely to be influenced by weak informational cues).

Our findings also suggest that social information in the form of trending information can exacerbate turbulence in political mobilization [10]. The most popular petitions receive more signatures than one would expect just by virtue of their popularity, and in this zero-sum race for collective attention, those for which there is no evidence of popularity receive fewer signatures than their popularity would indicate. In this way we have observed for political behaviour similar effects to those observed for cultural markets [30, 31], where experimental subjects were shown varying information about the popularity of otherwise unknown songs and were asked to rate them. Subjects were more likely to rate highly those songs for which there was evidence of popularity, meaning that the information injected a source of instability into cultural markets.

As mentioned already in reference to the two-column layout, small changes in the display of social information and the user interface may have significant impacts (as has been found for the display of comment threads [57]). The UK petition platform only let users sign petitions one at a time, and users had to re-enter their details (name, postcode, email address) separately for each petition they wished to sign. Specific deadlines, different calculations to determine which petitions are shown as trending, and other changes may weaken or strengthen the concentration of signatures.

We expect that this finding could be generalized beyond petition signing to other forms of political participation or support for political parties, as these kinds of popularity indicators are present in some form by default on most social media platforms. On Twitter, for example, it is possible to see immediately how many followers someone has, how many times a tweet has been liked or retweeted, and view trending topics. On Facebook, any post will show information about the number of ‘likes’ or other ‘reactions’ it has received, and global trending news is also shown. YouTube videos show the number of views and subscribers, and so on. It could be that these platforms—to greater or lesser extent according to their designs—are thereby contributing to the apparent rise in populism across Europe, the US, and other countries. It remains an open question for further research, as is the question of whether new platforms with very different presentations of social information (such as Snapchat, which does not show the number of followers or friends) create different effects and thereby feed into different distributions or whether people have other ways of working out social information from these platforms.

The importance of social media in disseminating petitions was illustrated through analysis of the analytics data we used in the previous section, which showed that around two thirds of all visitors to the petitions site arrived via either Twitter or Facebook. Further research could investigate the influence of the design of these alternative platforms, and how social information is presented within those designs, on the likelihood of petitions being disseminated and, ultimately, signed. Future work could compare the dissemination of petitions on Twitter, Facebook, or Instagram to their dissemination on a platform where there is little social information, such as Snapchat (which is increasingly popular amongst younger age groups). Such research would complement work that examines the connective appeal of Internet interactions [58] or explores the discursive potential of petition sites through design (e.g., [59, 60]). However, the closed nature of many of these platforms presents a serious challenge due to the lack of data available for research. The ease of obtaining data from Twitter, which has an open Application Programming Interface (API), is why this platform receives disproportionate levels of scholarly attention.

These findings have policy implications. The digital team in the Cabinet Office that developed the petition platform believed that the design change that introduced trending information would bring more people to sign petitions, but would not make any difference to which petitions they signed. In fact, the reverse was true, which indicates the importance of understanding the impact of design on behaviour for platforms geared at civic engagement. As discussed above, social information has been shown over decades of social science research to be an important driver of participation (see [10] for a review); so, we expected that if it was changed, behaviour would also change, but policy-makers will not necessarily be social scientists. This finding also illustrates the importance of testing design changes before they are fully implemented. Private firms offering services and products online—and indeed social media platforms themselves—continually run A/B tests, whereby different versions of a website are offered randomly to users and the effects on user behaviour measured and observed. Although officials we spoke to mentioned A/B testing was a tool being used within the Government Digital Service, it was not used for this particular design change.

The observation that some people come to the petition platform looking for something to sign is a further indicator of the importance of social information. These ‘aimless petitioners’ will be even more susceptible to whatever information or signals are presented to them on the platform than people directed to the site via links to specific petitions. Signing a petition on a digital platform is just one example of a ‘tiny act’ of participation (see [10]). These micro-contributions require less resources than any traditional act of participation and are drawing citizens with different demographics than traditionally assumed as politically active into mobilizations (for example, people from lower socio-economic groups, the ‘time-poor,’ and those with less interest in politics), which corresponds to other findings in this field [14, 15, 39]. Their decisions about which petitions to sign will be more easily shaped by information cues than those who come to the site looking for specific petitions or issues. Tiny acts may be amplified later as making small Internet-based contributions to political causes seems to lead to more substantive contributions (for example in the Obama election campaigns [61]). These findings make understanding the mechanisms that shape tiny acts (such as platform design) even more important.
